# 

**Published:** 1889-05

**Authors:** 


					﻿MAY 1889,
OLD SERIES
VoL XXV
NEW SERIES
Vil. VI —N > 3'
& 1855 0


rfHURNAfc



W. F. WESTMORELAND, M D
H. V. M. MILLER, M, D. LL, D.
VIRGIL 0, HARDON, M. D
^bushed By james rharrison&cq^
BP ____________Atlanta, ga. Ji
SUBSCRIPTION I.S2.BO A YEAR
				

